# Ward social workers’ views of what facilitates or hinders collaboration with specialist palliative care team social workers: A grounded theory

**DOI:** 10.1186/s12904-017-0214-z

**Published:** 2017-07-14

**Authors:** Janice Firn, Nancy Preston, Catherine Walshe

**Affiliations:** 1 Division of Geriatric and Palliative Medicine, Michigan Medicine, F7902 UH South, 1500 E. Medical Center Drive, SPC 5233, Ann Arbor, MI 48109 USA; 2 Division of Health Research, C52, Furness Building, Lancaster University, Bailrigg, Lancaster LA1 4YG UK; 3 International Observatory on End of Life Care, Lancaster, LA1 4YG UK

**Keywords:** Palliative care, Cooperative behaviour, Social work, Grounded theory, Qualitative research

## Abstract

**Background:**

Inpatient, generalist social workers in discharge planning roles work alongside specialist palliative care social workers to care for patients, often resulting in two social workers being concurrently involved in the same patient’s care. Previous studies identifying components of effective collaboration, which impacts patient outcomes, care efficiency, professional job satisfaction, and healthcare costs, were conducted with nurses and physicians but not social workers. This study explores ward social workers’ perceptions of what facilitates or hinders collaboration with palliative care social workers.

**Methods:**

Grounded theory was used to explore the research aim. In-depth qualitative interviews with masters trained ward social workers (*n* = 14) working in six hospitals located in the Midwest, United States were conducted between February 2014 and January 2015. A theoretical model of ward social workers’ collaboration with palliative care social workers was developed.

**Results:**

The emerging model of collaboration consists of: 1) trust, which is comprised of a) ability, b) benevolence, and c) integrity, 2) information sharing, and 3) role negotiation. Effective collaboration occurs when all elements of the model are present.

**Conclusion:**

Collaboration is facilitated when ward social workers’ perceptions of trust are high, pertinent information is communicated in a time-sensitive manner, and a flexible approach to roles is taken. The theoretical model of collaboration can inform organisational policy and social work clinical practice guidelines, and may be of use to other healthcare professionals, as improvements in collaboration among healthcare providers may have a positive impact on patient outcomes.

## Background

Understanding professional collaboration amongst health and social care providers is crucial as it is a vital part of achieving better patient outcomes, improving patient satisfaction, reducing length of stay, lowering costs, and contributing to fewer and shorter delays in the provision of care [1–8]. professional collaboration is central to the philosophy and provision of specialist palliative care consultation teams, which includes combining multiple health and social care professionals’ expertise to meet the needs of hospitalised patients and their families (see Table 1 for key terms) [9, 10]. The specialisation of palliative care and how to collaborate well with each other has been much debated by generalist and specialist nurses and physicians [11–18]. With the inclusion of a palliative care social work role on the hospital-based palliative care consultation team the generalist-specialist debate has come to the forefront for clinical social work [11–14, 16–18]. As each health or social care profession views collaboration from a different perspective, what one profession identifies as the factors most strongly contributing to collaboration may vary from those of another profession [19]. For social work collaboration to be successful it is important to understand what social workers view as collaborative. Very little is known about ward social workers’ perceptions of collaborating with palliative care social workers [20, 21].

**Table 1:** Key terms

• Hospital-based palliative care consultation teams do not assume the care of the patient. They offer advisory and advocacy services to patients, families, and staff to complement the services provided by the ward team, through delivering symptom control, psychosocial care, and end-of-life care for hospitalised adults [66, 67].
• ‘Generalist’ is defined as ‘the acquisition and application of a broad spectrum of knowledge and skills (p. 141) [18]’ that can be used to address the range of different situations regularly encountered caring for patients in the hospital. Here, generalist is used to refer to health and social care providers who are not part of the specialist palliative care team, such as oncologists, neurologists, and the ward social worker
• ‘Specialist’ in this context refers to the specialist palliative care team members, including the specialist palliative care social worker, who have ‘superior knowledge and skill acquired through extensive practice experience and/or additional training (p. 142) [18]’ in palliative care

Social work as a profession places a strong emphasis on teamwork and collaboration [22]. Over time, hospital-based social workers have worked to develop their role on the interprofessional healthcare team, resulting in a strong sense of ownership regarding their work [17, 23]. Prior to the development and growth of palliative care, generalist ward social workers provided the full range of end of life services [24]. Today, for hospitals which employ both generalist ward social workers and specialist palliative care social workers, more than one social worker may be involved in providing care to the same patient. In principle, each social worker has similar education, professional training, and share the same status within the institution; they are peers within the organisational hierarchy. In these situations, there is concern that social workers’ similar education, skill set, and organisational standing could lead to role confusion and challenges in care delivery. These issues have not been studied in the hospital setting.

Greater understanding of the underlying factors contributing to ward social workers’ perceptions of collaboration could explain the conditions under which collaboration is facilitated or hindered. This information could assist with the development of professional social work practice, which could positively impact healthcare quality and patient outcomes. Therefore, this study explores and develops a theory of ward social workers’ perceptions of what facilitates or hinders collaboration with palliative care social workers in the hospital.

## Methods

### Qualitative approach and research paradigm

Critical realist grounded theory was used to explore ward social workers’ views of what facilitates or hinders collaboration with palliative care social workers. Critical realist grounded theory takes into account the event being studied (collaboration), the individual meanings made of it (social workers’ perceptions), and the broader social structures (hospital setting and professional training) and the generating mechanisms behind the event [25–27].

### Researcher characteristics and reflexivity

The researchers have familiarity with palliative care; JF is a palliative care social worker in the United States, NP and CW work in England and have nursing backgrounds. The researchers do not work at hospitals where the research was conducted. Careful reflection and acknowledgement was given to the researchers’ participation in theoretical development. Reflexivity was utilised to reduce bias and avoid previous knowledge interfering with new insights into the data.

### Context and sampling strategy

Masters trained social workers from hospitals located in the state of Michigan in the Midwest, United States were recruited to participate in the study. Included in the study were English speaking ward social workers working with adult patients (patients 18 years old and older) in both for-profit and not-for-profit hospitals with palliative care consultation teams which included a palliative care social worker. Social workers in military and children’s hospitals, or working at hospitals without a palliative care social worker, social work students or those not masters trained (i.e. those with a bachelor of social work degree), and palliative care social workers were excluded from the study.

Social work directors at hospitals in Michigan with palliative care teams which included social workers, identified through the National Palliative Care Registry [28], were approached for permission to recruit social workers from their departments. The hospital where the researcher works as a palliative care social worker was excluded from the study, leaving 12 hospitals from which to recruit potential participants. Six directors did not respond to attempts to contact them. For those that responded, once permission was obtained from the department directors, who provided ward social workers’ email addresses, potential participants were contacted directly by JF via email. Theoretical sampling was utilised. Initially, recruitment was open but became more focused over time guided by theoretical sampling. To meet the needs of theoretical sampling and reach theoretical saturation diversity was sought in social work experience, disease type, age, location, and number of years postmasters training. Diversity was also sought in hospital type (academic vs. private vs. public), size (number of beds), and location. The intent of seeking this diversity was not to obtain a representative sample or to increase generalisability, rather, through theoretical sampling, to fit the emerging theory to the data. Non-participating hospitals had similar characteristics to participating hospitals. The total number of ward social workers at the non-responding and participating hospitals is unknown. It is not possible to determine how many social workers declined to participate.

### Data collection

To explore ward social workers’ experiences of collaborating with the palliative care social worker qualitative interviews were used. In-person interviews were conducted and coded by JF in discussion with NP and CW. Data was de-identified prior to sharing with NP and CW, and throughout the reporting of the data. Participants were not explicitly informed of JF’s role in palliative care. Interviews were carried out at participants’ workplaces, digitally recorded, and were conversational in style using open-ended questions from an interview guide. An iterative, reflexive approach was taken throughout the interview process; the interview guide questions changed and developed over time. Interviews lasted from 17 to 53 min, with a median time of 27 min. Data collection ceased when theoretical saturation (described below) was reached. Interviews were not returned to participants.

### Data processing and analysis

Interview recordings were transcribed and analysed by JF in discussion with NP and CW to enhance the trustworthiness of the data, for emerging key themes using Charmaz’s [29] grounded theory technique. Charmaz’s [29] underlying philosophical stance is constructivist rather than realist; however, she notes that the grounded theory analysis steps outlined in her book are compatible with other philosophical stances. Thus, operationalising the techniques outlined in her book from a critical realist stance is conceivable [25–27]. NVivo (version 10) was used to organize the data and uphold rigour through data tracking [30]. Concurrent data collection guided by theoretical sampling and analysis occurred throughout the course of theoretical development. An iterative analysis process was applied. Interviews were analysed as they were completed, allowing each proceeding interview to be informed by those which preceded it. Theoretical sampling ceased when theoretical saturation occurred. Theoretical saturation was considered to be met when the identified themes were robust and no new codes emerged from the data [29]. The quality and sufficiency of the data and the level of thematic saturation were assessed from a critical realist lens and by using criteria proposed by Charmaz [29], which evaluate whether enough background data about persons, processes, and settings is available to allow researchers to understand and portray the full range of the context of the study, as well as identify causal mechanisms and facilitators of collaboration. The researchers spoke regularly to discuss coding, analysis, and data interpretation, and to examine the ways in which professional vantage points and previous knowledge could bias theoretical development.

## Results

Masters trained social workers (*n* = 14) working in the hospitals in Michigan who share cases with palliative care social workers were recruited from February 2014 through January 2015 to participate in the study. They represented all areas of the adult inpatient wards (Table 2).

**Table 2:** Participant and hospital characteristics

Participant characteristics (*n* = 14)	
Sex:	
- 14 Female^a^	
Race:	
- 13 Caucasian	
- 1 African American	
Age (*n* = 14):	
- 25–55 years, median 40 years old	
Years post MSW training:	
- 3–32 years, median 12.5 years	
Patient case load:	
- 20–50 patients per social worker, median 36 patients	
Frequency of contact with palliative care social worker:	
- >1 month – daily, average 2–3 times a week	
Length of time palliative care team active in hospital:	
- ≥ 5 years	
Hospital characteristics (*n* = 6)	
Hospital size:	
- 300–1100 beds, median 640 beds	
Hospital location:	
- 2 Small community areas	
- 2 Larger urban areas	
- 2 Inner-city	

^a^Social workers in the U.S. are mostly female (82%); sample homogeneity is not unexpected [68]

### Theory of collaboration

**Fig. 1:**
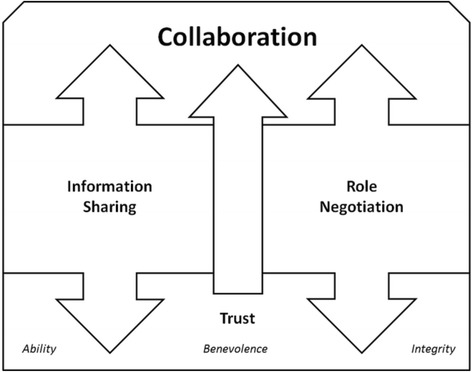
Theoretical model of ward social workers’ collaboration with palliative care social workers

Data analysis resulted in the development a grounded theory of ward social worker’s collaboration with palliative care social workers. Collaboration consists of three constructs: *Trust*, *Information Sharing*, and *Role Negotiation* (Fig. 1). Trust has three components: *ability*, *benevolence*, and *integrity*. All three components need to be present for Trust to occur. Positive interactions in role negotiation and information sharing can strengthen trust. However, if trust is lacking true role negotiation and information sharing cannot take place. Even when trust is present, ward social workers’ collaboration with palliative care social workers does not happen if the interaction lacks either effective information sharing or effective role negotiation. All three constructs need to be in place for collaboration to take place. Each construct is discussed in separate sub-sections below. Quotations are used to illustrate the data (Tables 3, 4, 5, 6 & 7). Participants are identified by the letter “P” and a number (1–14).

### Trust

Trust is essential to collaboration. It is comprised of three parts: *ability*, *benevolence*, and *integrity.*

### Ability

*Ability* is the first component of Trust. Provision of palliative care services was not thought by ward social workers to require specialty training or superior expertise. Rather, ward social workers expressed that any social worker with the desire, time, practice, experience, and opportunity to develop a palliative care related knowledge base could provide services at a similar level to the palliative care social worker.

*“We’re kind of expected to deal with a wide range of things, and I don’t know if there’s anything specific that she does that we absolutely couldn’t do… she would maybe be more experienced, more comfortable, more confident.”* – P10.

Instead, ability refers to the ward social workers’ perception of the specific clinical skills, team management competencies, and amount of experience needed to provide palliative care social work services. Perceptions of the palliative care social worker’s abilities impact the ward social worker’s willingness to collaborate.

*“[For collaboration] trust is important about her clinical skills and abilities.*” - P1.

Ward social workers perceive competent palliative care social workers to have the necessary skills to manage the ward team’s dynamics and needs, remain in a consultative role, and provide expert resources and recommendations to meet the complex needs of patients and families. These skills include: facilitate goals of care conversations, bereavement support, resource knowledge, and awareness of how to transition from standard care to palliative care, and from palliative care to hospice. Beyond these skills, ward social workers also think palliative care social workers need a good understanding of medical terminology and disease processes in order to properly guide patients and families. When the palliative care social worker is perceived to be a strong, capable clinician, trust is high and collaboration is facilitated. Conversely, perceptions that the palliative care social worker lacks ability and is a weak clinician lead to mistrust.

*“She doesn’t really have a case load… I would’ve defined the role a lot different than what she’s doing. She’s sitting back and getting directions from the nurse practitioners and the physicians in the group as opposed to stepping out and defining her own role. So she doesn’t get consults… I think [she] has the time and opportunity to follow up on cases but [she] doesn’t understand what to do in a follow up”* - P6.

When trust is impaired collaboration is deterred.

**Table 3:** Ability additional supporting quotes

• *“[Palliative care social worker] is dealing not only with family dynamics that are very intense, and cultural issues … that come up quite a bit in hospice, [she] has to have that medical background to explain what’s going. And then, of course, knowing when to call the doctor if they have specific other questions. It takes a little bit of training and experience.”* – P7
• *“… it takes a skill base, and as you know, you build on your skill base with experience, and knowing the different kinds of venues to approach a family, how to asses a family, and knowing what to say, what not to say, how to say it, even your tone of voice and how you speak to them, how you enter then room. All of those things are very important when you’re dealing with families and working with families for end-of-life. So, I would agree with the skill base, I wouldn’t say specifically trained, because only experience trains you for something like that*.” – P11
• *“Every social worker should be able to provide palliative care and end of life services. As long as you’re comfortable with death and dying and really sick patients, you should be able to do this for sure.”* – P12
• *“But part of why we have different teams, different [social work] specialties is that I’m not going to know everything.”* – P4
• *“You can transfer a lot of your skills to a situation that’s dealing with end of life, but the palliative social worker is going to be a lot more familiar with that, whereas I’m going to be more familiar with something else, like talking to kids about their parents, and ICU. So, that palliative social worker, they could figure that out, you know what I mean? They could do that, but I just might be more readily able to do it.”* – P10

#### Benevolence

*Benevolence*, the second component of Trust, relates to the ward social worker’s perception that the palliative care social worker has her best interest at heart. Ward social workers identify palliative care social workers as demonstrating benevolence towards them when the palliative care social worker is helpful, supports the ward social worker’s role, respects her skills, and shares the burden of caring for the patient and family at end of life.

*“I respect [her] expertise and [she] respects my expertise on what I get done and what can I do to help the patient and the family.”* – P3.

The palliative care social worker demonstrates a ready desire to help by being easy to contact and communicate with, and quick to respond to needs. Limited (or no) interaction by the ward social worker with the palliative care social worker prohibits relationship building. The lack of relationship reduces the perception of benevolence, decreasing feelings of trust, which in turn hinders collaboration. The perception that benevolence is lacking creates a territorial response on the ward social worker’s part.

*“I would like a little bit more hands-off approach and understand that I do what you do too. I'm the social worker on this unit. There’s a reason why I'm in this position… So, I'm a little bossy. I'm a little assertive… These patients are my babies. All of them are. It starts and stops in this office but it starts and stops with me first.” –* P5.

Instead of working towards collaboration, possessive and defensive behaviours occur. Trust is additionally damaged when the palliative care social worker implies to patients that the ward social worker will do something for them that cannot happen.

*“There’s a limited understanding of what the ward social worker does. And so then there are these promises that are made that the ward social worker can do x,y,z.”* – P4.

This behaviour on the part of the palliative care social worker is seen as disrespectful and un-collaborative. The lack of benevolence undermines trust and collaboration.

**Table 4:** Benevolence additional supporting quotes

• *“She’ll come up that same day and meet with the patient and the family and contact family wherever they are.”* – P3
• *“I’ll call her sometimes with a scenario, or if she’s met a family, I’ll run something by her… so she’s been really available in that way, so that’s ideal for me too, as a resource.”* – P10
• *“As long as there’s a proactive [approach] in terms of everybody kind of being on the same page for the care of the patient, yes it’s good for the care of the patient, and continuation of care and yada yada, but it also just makes it easier for everybody.”* – P4
• *“Getting to know that social worker [having a relationship helps with collaboration]”* – P10
• *“The palliative care social worker is my frontline person since we have the same kind of job … we’ve created this relationship … that has given us some mutual understanding of how we work together.”* - P2
• *“The social worker, she mainly stays down in the ICU. So I do not see her much.”* - P3
• *“Just having more... a little bit more engagement about what her thought process was, I think, that that would’ve been very helpful, had I gotten more from her.”* – P14
• *“Understanding what your role is in the case, understanding what [her] role is in the case and being very clear about that.”* – P2

#### Integrity

*Integrity*, the third component of Trust, centres on the needs of patients and families. Ward social workers trust palliative care social workers who demonstrate integrity. Palliative care social workers are viewed as having high integrity when they adhere to the principles of patient-centred care delineated in the professional ethics of social work [31, 32]. The first principle is a commitment to patient empowerment, ensuring that patients have all the information needed to make the informed decisions.

*“[Help the patient understand] what's really happening…what the long-term impact is… what is this gonna look like, six months down the line… to help the patient to understand, in English, what it means.”* – P14.

The second principle is honouring patient autonomy and self-determination. This principle supports the concept that competent adults are able to make their own decisions about what is in their own best interest, even if the social worker does not agree with the decision.

“*Thinking about what’s best for the patient, and the family*.” – P11.

The third principle is that each patient be treated individually. This principle flows from the social work value of viewing the patient as an entire person, mind, body, and soul, within the context of their relationships, environment, financial situation, and practical limitations. Palliative care social workers are perceived to lack integrity when they seem to be forcing a patient to do something that is in the best interest of the hospital rather than the best interest of the patient.

“*Looking at hospital policy more than patient care*… *If the palliative care social worker worked with the values that I see that are important, absolutely I would want her involved in more cases.*” - P6.

When the palliative care social worker is perceived to value institutional needs above those of the patient, she is viewed as an agent of the institution rather than an advocate for the patient. The ward social worker does not trust or want to work with the palliative care social worker who fails to uphold patient-centred values. Lacking integrity, therefore, is a barrier to collaboration.

To summarise, when the ward social worker perceives the palliative care social worker to have high *ability*, *benevolence*, and *integrity*, belief in her trustworthiness is increased, which facilitates collaboration.

**Table 5:** Integrity additional supporting quotes

• *“You should really treat each person individually”* – P1
• *“But my goal with my patients is—and my motto is ‘You cannot make a good decision unless you’re fully informed.’ I just try to make sure that they know everything, that they know the repercussions that they may be getting into. And then, honestly, if they’re fully competent in making their own decisions and they want a further treatment, I’m not going to stop them. That’s what they want to do. I’m here to advocate and support for them.”* – P8
• *“Because palliative care in an ideal setting… would be gathering and assessing with the patient what their goals are and then trying to help meet those goals… and that might be continued therapy or it might be go home with a hospice.”* - P13
• *“…ultimately at the end of the day, it’s all about the patient. If the patient’s not getting what they need, I’m not happy.”* – P5
• *“I mean, even though we’re not really doing much for the patient. We still allow them that time to, I guess, adjust to the idea.”* – P9
• *“…for palliative care there was sort of a financial … almost obvious to me that they were pushing getting people out of the hospital and never staying [to save the hospital money].”* – P13
• *“If you have a family that’s not ready to make that decision, and you keep approaching them with it. That’s when it complicates things, because we have to respect when a family’s not ready for that, because if we keep pursing, and pursing, and pursing, it impairs their coping, and then that disables their decision-making.”* – P11

### Information sharing

The next construct of collaboration is Information Sharing. Ward and palliative care social workers share information about cases through formal and informal verbal communication, in person or by phone, or through direct written communication via email or text-paging, as well as reading what is documented in the electronic medical record.

*“We do a lot of paging back and forth. Sometimes e-mails but mostly phone conversations, “hey! I found out this”” –* P2.

Ward social workers desire that shared information consist of family dynamics, patient and family system coping, and what the palliative care social worker’s role will be in the case.

*“[Give information] on the nature of what the patient is in the hospital for, the complicated medical course, what’s been going on… the key players, and the patient’s decision making… what have you been communicating.”* - P11.

If the ward social worker was not present for specific conversations good information sharing by the palliative care social worker includes providing a summary of what occurred during the palliative care teams’ interactions with the patient, family, and ward team.

“*[Palliative care social worker] just spent an hour with the family*… *Communication is a really important aspect of it; I need to know what I might be walking into… If family members are not on the same page, there will be problems.”* – P9.

For communication to work well it must be two-way, timely, and consistent. Ward social workers report that collaboration is served when the ward and palliative care social workers take responsibility to each own communication and proactively communicate with one another.

“*I think that communication is key [as a best practice] … and trying to make sure that everyone’s on the same page.”* – P7.

Poor communication results in duplication of services, redundant work, or inability to meet the needs of patients and families. Poor communication may also over-burden patients and families with the ward social worker asking the same questions just covered by the palliative care social worker, or vice versa.

*“And the patients are like, “This is stupid. What’s happening? I already told someone this.””* – P8.

When communication is lacking, delayed, inconsistent, or perceived as burdensome to the patient and family collaboration is obstructed.

**Table 6:** Information sharing additional supporting quotes

• *“When we have our [team] conferences… best practice would be to be able to have everyone involved in the case there so that it’s coordinated* versus *feeling somewhat disjointed.”* – P1
• *“Best practice would definitely be more collaboration and notification before [palliative care social worker] is involved… there’s more to it than just what’s in the documentation.”* – P5
• *“A little bit more engagement about her thought process … sometimes I wouldn’t get the feedback I needed… I knew she was in there but if there was something specific she was working on I wouldn’t always know what that was... There was a little bit of the lack of communication and most of the communication was initiated on my part. So, if there had been a little bit two-way, it would’ve been better.”* – P14
• *“You don’t want to have two and three different social workers [going in and out of the room asking the same questions] because families get very irritated when they have to constantly repeat themselves… so you want to limit that and limit their stress from anything.”* – P11
• *“I think having too many people in there just confuses families; it makes them very overwhelmed because people are telling them different things.”* – P9
• *“It is very important that everybody is saying the same things to the patient and family.”* – P3
• *“There are just so many involved when the patient’s here… I think there’s so many people involved that sometimes the communication is not good and so that makes [collaboration] really difficult.”* – P6

### Role negotiation

Ward social workers’ report no formal organisational differentiation between the ward and palliative care social work skill sets and roles.

*We always work it out ourselves [there is no institutional directive for roles].* – P12.

Thus, Role Negotiation, the third construct of the theory, is a key feature of collaboration. Ward social workers report they primarily manage discharge planning needs, while the palliative care social worker is responsible for participating in family meetings and patient care conferences to develop a plan of care related to end of life needs.

*“If we were doing a lot of [counselling] work, we would never get our discharge planning done*.” – P13.

Discharge planning takes precedence over other types of clinical work for ward social workers. They report feeling busy and stretched thin with little time, given their caseloads and job expectations, to address both the palliative care and discharge planning needs of the patient.

*“I wish I had as much time as they do but really [palliative care social worker] is in there because that’s what they're there for, that time to sit down, to digest it all with the family.”* – P8.

Additionally, the ward social worker may or may not have the desire for and required skills to provide palliative care services. Thus, comfort level also contributes to how roles are negotiated.

Negotiation decisions, in addition to being made based on time, comfort, and institutional priorities, are also made based on the therapeutic relationship the ward social worker has with the patient.

*“If I’ve been working with the family and I have a relationship with them, I’m right on the unit… it’s like a case-by-case of how we go for it… if it’s a family that I had no involvement with, then usually she is the one that follows, if that makes sense. So, it’s really, there’s not really a process, it’s more like with each family, we decide afterwards.” –* P10.

Ward social workers value the relationships they have with patients and families, and desire for the palliative care social worker to respect pre-existing relationships, and the ward social worker, by supporting the continuation of the relationship. When a long-standing therapeutic relationship is in place the relationship may be the determining factor for which social worker plays which role.

With little organisational direction for either role, the challenge of time constraints, and pressures that arise from institutional mandates to facilitate discharge, a willingness to be flexible about which social worker does what task is essential for effective role negotiation. Flexibility is possible with trust. The lack of trust makes flexibility in role negotiation less possible and undermines collaboration.

*“We don’t negotiate who does what. We don’t negotiate because at the end of the day, on this unit, it starts and stops with this office.”* - P5.

The palliative care social worker may still be involved in the case but the ward social worker may actively block or undermine the palliative care social worker’s role. When role negotiation does not take place or when it fails, collaboration cannot be achieved.

**Table 7:** Role negotiation additional supporting quotes

*• “I wish I had as much time as they do but really [specialist palliative care social worker] is in there because that’s what they’re there for, that time to sit down, to digest it all with the family.”* – P8
• *“If we were doing a lot of [counselling] work, we would never get our discharge planning done.”* – P13
• *“Having 40 patients on a [ward] gets really difficult sometimes... Sharing care with the palliative care social worker I see it as a positive. I mean it helps me out in my role… It saves me time. It saves me energy.”* - P9
• “Some people like just doing discharge planning, some people like myself like to have a variety of things to do during the day.” – P7
• “The one area I always wanted desperately to avoid was death and dying.” – P1
• *“I don’t know how [we figure out which social worker does what], I think it depends on the social worker, like I know one of us, once palliative gets involved, she prefers [palliative care social worker] take over.”* – P10
• *“Every patient that crosses my path, it’s not about me. It’s never about me. It’s always about the patient… whatever needs to happen happens and it’s okay if it’s not me that’s providing it.”* – P1
• *“Why are you doing that family meeting? Why are you doing that? I can do that.”* – P8
• “Flexibility is important… I’m more than willing to negotiate and back and forth, I want her to *want* to come and work with me and work with my patients versus “oh gosh. Here’s another one that I’m going to have to take over and I don’t have the time to do it.”” - P1

## Discussion

This is the first study to explore ward social workers’ perceptions of collaboration with their palliative care social work peers. The key constructs of collaboration are: Trust (comprised of *ability*, *benevolence*, and *integrity*), Information Sharing, and Role Negotiation. When all three constructs are in place and operating well, the ward social worker perceives interactions with the palliative care social worker as collaborative. When one or more pieces are missing the ward social worker does not experience interactions with the palliative care social worker as collaborative. This theory may have applicability beyond social work for other healthcare providers when there are two professionals of similar training and background involved in a patient’s care at the same time, one in a primary role and the other involved episodically. Each component is discussed in a separate subsection below.

### Trust

Trust is well supported in the literature as a key component of collaboration [33, 34]. The emerging theoretical understating of trust here most closely reflects the “*Integrated Model of Organizational Trust*” developed by Mayer et al. (1995) [34]. They also posited that trust is comprised of *ability*, *benevolence*, and *integrity*. However, Mayer et al. (1995) [34] focus on the role of trust in hierarchal relationships as work, between supervisor and supervisee. The results from this study validate and support Mayer et al.’s (1995) [34] theory. What is more, these results add substantially to their model by establishing *ability, benevolence*, and *integrity* as key components for trusting peers. Thus, the results from this study enhance and expand the previous theoretical understanding of the role of trust in relationships at work.

#### Ability

Other theorists, in addition to Mayer et al. (1995) [34] have also identified *ability* as a key component of trust [35–37]. These prior studies, again, focused on trust between superiors and subordinates where there is a clear, organisationally defined power differential between the two parties [34]. The results here add to the existing literature; indicating that even when no formally defined power differential exists, perceptions of ability are still important for collaboration with peers [33, 38, 39].

Ability can be defined as the group of skills, aptitudes, and characteristics that allow a person to have expertise within a specific area [34]. Expertise in one area is not thought to be generalisable to another [40]. Social workers develop the competencies needed to be experts in a specific domain by working in that domain [18, 41]. Expertise occurs when social workers have had time to cultivate a professional working style, internalise theory and research, develop a way to measure success, and shed pieces of the professional role which are incongruent with the self [41]. Not every social worker will have the time, inclination, or opportunity to gain expertise in palliative care [18, 41]. For those who do have the desire and inclination, the current organisational needs and primacy of discharge planning may mean they have no opportunity to develop these skills. Lacking these skills may result in patients and families having unmet needs in settings where there are no palliative care social workers.

Lastly, within generalist social work practice advanced levels of knowledge and skill can be acquired [18, 41]. Ward social workers can be ‘experts’ in their own right by having specific knowledge and skills related to hospital discharge planning. The scope of practice, skill set, and experience needed for discharge planning differs from that of the palliative care social worker [24, 42, 43]. In order to meet the complex needs of patients both the ward and palliative care social work roles are important for the delivery of quality patient care.

#### Benevolence

*Benevolence* as a component of trust is also well supported in the literature [34, 44–46]. Again, Mayer et al.’s (1995) [34] understanding of benevolence was developed as a result of studying relationships between managers and line staff. This study adds to the theoretical understanding of the role of benevolence by highlighting its role in trusting peers. It also further supports previous findings on both the importance of professional relationships and their positive impact on perceptions of collaboration [33, 47, 48]. A study of community palliative care nurse and physician providers found that developing strong relationships facilitates perceptions of cohesion and contributes to wards’ ability to do their own work, this study confirms similar patterns of relating occur in the inpatient setting with social workers as well [49]. Additionally, similar to previous studies, relationships and collaboration were enhanced by co-location [12, 21, 49, 50]. The similarities between these results and existing knowledge, which primarily stems from studies of nurses and physicians, are noteworthy. These findings may be applicable across professions, which could help further clinical practice for other professions, and could lead in the future to a more overarching generalisable theory.

#### Integrity

*Integrity* is the third component of Trust. Mayer et al. (1995) [34] reported that integrity between superiors and subordinates involves each adhering to a set of values that the other finds acceptable. This study adds to the exiting theoretical understanding of trust by identifying the role of integrity in trusting peers. In the literature, as in this study, collaborative professional relationships include trusting that all team members are working for the common good of the patient and family [38, 51–53]. These findings have implications not only for palliative care social workers but for all members of the interprofessional palliative care team. From an organisational standpoint, particularly in the United States, palliative care teams are often viewed as a way to save the hospital money by decreasing length of stay, preventing admissions, and limiting costly interventions [54]. Palliative care teams, in principle, share values similar to social work, seeking to empower patients and families to make autonomous decisions. It seems from ward social workers’ responses that there is room for improvement for both palliative care teams and palliative care social workers to ensure they are adhering to the values they profess. Palliative care teams must vigilantly maintain their focus to provide patient-centred care, otherwise they risk losing their purpose and discouraging other health care professionals from collaborating with them.

### Information sharing

These results are consistent with findings from the broader literature on the centrality of communication in effective interprofessional collaboration. In interprofessional interactions sharing information consists of verbal, written, and non-verbal communication between team members and is demonstrated through listening, negotiating, consulting, interacting, discussing or debating with one another [51, 53]. More importantly, the findings show that the elements of good communication are similar for uni-disciplinary and inter-professional interactions. Like interprofessional teams, ward social workers from this study report experiencing higher levels of collaboration when they are given regular, formal opportunities to communicate, as well as have informal opportunities for communication [33, 51, 52]. The similarities between interprofessional and unidisciplinary team communication allow for knowledge about what improves and facilitates one type of interaction to be applied to the other.

As a profession social workers view themselves as being ‘good communicators’ [55]. The same is true for palliative care teams, who are often called upon to be involved in situations of difficult team, patient or family dynamics because of their communication skills [56]. However, there is evidence from this study and others that despite social work training or palliative care team membership there is room for improvement in communication [57, 58]. It is not enough to be a ‘good communicator’ with patients and families, these skills must be carried into interactions with other staff members as well [59]. Careful attention to communication will go far to facilitate collaboration.

### Role negotiation

Ward social workers reported a lack of organisational clarity for their job responsibilities and skill sets, leading to role confusion and the need for role negotiation. The importance of organisational direction in determining roles and facilitating collaboration for interprofessional teams is well supported in the literature [33, 38, 51]. The need for organisational direction appears in intraprofessional interactions as well. However, because of the diversity of tasks and the unpredictability of patient and family care needs, ward and palliative care social workers may still need a degree of flexibility in their roles to respond to each case individually [12]. A more defined job description at an organisational level could be helpful for facilitating collaboration, as long as it allows for some flexibility, by adding additional functionality and efficiency to the ward and palliative care social work roles.

Additional organisational guidance may also help with determining which social worker is best suited skill-wise to address specific needs of the patient and family. Ward social workers spoke of using their therapeutic relationships with patients and families to approach negotiating social work roles. This tactic assumes that patient-provider familiarity best facilitates the provision of psychosocial support, prevents additional burden, and reduces confusion. Previous studies regarding the significance of relationships for addressing psychosocial needs, like this study, were mainly based on self-report by the clinician and did not study clients’ perspectives, potentially limiting the usefulness of the data for adequately informing care decisions [60–64]. A more recent study of nurses found that an established relationship is not necessary for the provision of psychosocial support, an established relationship does not guarantee that it is effectively therapeutic for appropriately addressing psychosocial needs, and patients perceive relationships with providers differently from their providers [65]. Whilst Hill’s (2014) [65] study was conducted with nurses, it raises concerns for how social workers’ assumptions about relationships may be influencing role negotiation. Ward and palliative care social workers need to be aware of this potential bias when determining which social worker participates in palliative care conversations. The social worker with the skill set and knowledge base to best meet the patient’s needs is the appropriate person to address them.

### Limitations

There are several limitations to the study. Participants are from the United States, located in only one state, predominately Caucasian, and all women. The theoretical model may change or expand if respondents from other states, countries, ethnicities, and cultures were included. The addition of male respondents may also alter the theoretical model. Recruitment may have limited the results as well. Attempts were made to contact a number of hospitals and social workers; not all responded. As social work managers served as gatekeepers for accessing the line staff, they may have introduced bias by the way they identified or requested participants. Some potential participants may have been excluded. Participants who had a predominantly positive or a predominantly negative view of collaborating with specialist palliative care social workers may have chosen to participate, thereby skewing the data in a particular direction. Finally, despite using reflexivity to minimise the risk of bias, bias may have occurred.

### Strengths

The study rigorously adhered to the grounded theory methodology. Rich data were collected that allowed for the emergence of themes, which in turn made it possible to interpret the data and construct an original theory. An iterative approach was applied throughout to refine and develop the data collection, analysis, and results. Constant comparison, combined with discussions amongst the authors was utilised to reduce bias. These discussions contributed to more in-depth analysis. Having a variety of respondents representing a range of ages and work experiences, from several hospitals in different locations caring for different populations of patients, made it possible to do theoretical sampling and reach theoretical saturation. The constructs of trust, communication, and role negotiation described in this study have similar features to those found in previous studies. In the literature these elements of collaboration appear in a number of workplace environments and professions. Whilst the model of collaboration here is specifically derived from social workers’ in-hospital interactions in Michigan in the Midwest United Sates, because it shares commonalities with extant literature it may be applicable to a number of settings and types of healthcare professionals.

### Implications for practice, policy, and research

This study has implications for organisational policy, social work clinical practice, and future research directions. Further research is needed comparing both ward and palliative care social workers’ impact on patient outcomes. This information may give guidance to organisations and social workers about which social worker is best suited, skill wise, to positively impact patient outcomes, informing both organisations’ hiring practices and staffing decisions and social work clinical practice. Exploring patients’ perceptions of care and patient preferences, along with gathering more knowledge of what both ward and palliative care social workers uniquely bring to the patient encounter may also provide guidance for decision making about practice, roles, and division of responsibilities. Additional areas of future research include exploring palliative care social workers’ views of collaborating with ward social workers, and exploring ward social workers’ perceptions of collaborating with the interprofessional palliative care team as a whole. Finally, further exploration of the role of the three components of trust in peer-to-peer relationships is needed in other settings and with other professions. Addressing collaboration from these directions may alter the existing theory and contribute to a more global theory of collaboration. A more global theory of collaboration could be of benefit to a broader variety of professionals working in areas within and outside of healthcare.

## Conclusion

The novel, emerging model of collaboration consists of Trust, Information Sharing, and Role Negotiation. As a result of this study the components of ward social workers’ collaboration with palliative care social workers are better understood. This new awareness must inform clinical practice and policies at the organisational and practitioner levels. The barriers to collaboration identified here are not unique to the field of social work; they have been noted in the literature to be present in a variety of interprofessional interactions. Whilst similarities were surmised about social work intraprofessional relationships, now there is empirical evidence on which to base decisions. Social work can now draw more liberally upon and apply information about communication best practices and role negotiation from the existing interprofessional collaboration and teamwork literature to develop their own guidelines. Guidelines should address the roles of the ward and palliative care social workers within the organisation, division of labour between social workers, and provide recommendations for formal and informal communication. With attention to the development of clinical practice guidelines and an awareness of the role of trust in close-working peer relationships, social work collaboration will be more efficient and effective, which will ultimately have an impact on patient outcomes. Maintaining a consistent and high standard of care is good for the profession of social work, the organisations where they work, and for patients and families.
